# Severe Leptospirosis with Multiple Organ Failure Successfully Treated by Plasma Exchange and High-Volume Hemofiltration

**DOI:** 10.1155/2011/817414

**Published:** 2011-09-13

**Authors:** Vincent Bourquin, Belén Ponte, Bernard Hirschel, Jérôme Pugin, Pierre-Yves Martin, Patrick Saudan

**Affiliations:** ^1^Service de Néphrologie, Département de Médecine Interne, Hôpitaux Universitaires de Genève, 1205 Genève, Switzerland; ^2^Service des Maladies Infectieuses, Département de Médecine Interne, Hôpitaux Universitaires de Genève, 1205 Genève, Switzerland; ^3^Service des Soins Intensifs, Département d'Anesthésiologie, de Pharmacologie et des Soins Intensifs, Hôpitaux Universitaires de Genève, 1205 Genève, Switzerland

## Abstract

*Background*. Leptospirosis is a spirochetal zoonosis with complex clinical features including renal and liver failure. *Case report*. We report the case of a Swiss fisherman presenting with leptospirosis. After initial improvement, refractory septic shock and severe liver and kidney failure developed. The expected mortality was estimated at 90% with clinical scores. The patient underwent plasma exchanges and high-volume hemofiltration (HVHF) with complete recovery of hepatic and kidney functions. *Discussion*. Plasma exchanges and HVHF may confer survival benefit on patients with severe leptospirosis, refractory septic shock, and multiple-organ failure.

## 1. Introduction

Leptospirosis is a rare infectious disease in Switzerland. It is a spirochetal zoonosis caused by pathogenic Leptospira species. Human leptospiral infection results primarily from exposure to the urine of infected rats. Leptospires can then contaminate humans through cuts and abrasions of the skin, through intact mucous membranes (nose, mouth, eyes) and perhaps through waterlogged skin. The disease presents with complex clinical features varying from subclinical infection and self-limiting anicteric illness to multiple-organ failure and death [1]. 

In presence of kidney and liver failure, it is denominated Weil's disease. Antibiotics should be initiated as soon as the diagnosis is suspected [2]. In case of severe acute kidney injury, there is no consensus on the best method of dialysis, but the tendency is to propose the earliest and most intensive therapy available [3]. 

The disease can, in rare cases, evolve into refractory septic shock. In this paper, we report such a severe case and its management.

## 2. Case Report

A 65-years-old Swiss fisherman, known for hypertension, hypercholesterolemia, and gout presents with fever, epigastric pain, myalgia, and jaundice lasting for one week. Initial laboratory exam reveals acute kidney injury, liver failure, elevated troponin I, and thrombocytopenia (Table 1). Because of frequent arrhythmia (ventricular extrasystole) and an elevated troponin I raising the suspicion of myocarditis, he is admitted during 48 hours to the intensive care unit (ICU) for monitoring. 

Because of the initial presentation and the patient's occupation, leptospirosis was immediately suspected and treated with antibiotics (ceftriaxone). Initially, renal function improved. Serologies for *Leptospira icterohaemorrhagiae* were positive, confirming the diagnosis.

Suddenly, 7 days after the beginning of his hospitalization, he presented multiple-organ failure (MOF) of liver, kidney, and lung required mechanical ventilation and high doses of norepinephrine. His APACHE II and SAPS II scores were 37 and 80, respectively, which means an expected 90% mortality (see Table 1 for others parameters). 

Plasma exchanges were started, followed by continuous renal replacement therapy with high-volume hemofiltration (HVHF) at 70 mL/kg/h. He received five sessions of plasma exchanges and was treated during 72 hours by HVHF, followed then by 10 days of standard hemodiafiltration before renal function recovered. 

His ICU stay was complicated by a penicillin-resistant enterococcus bacteremia requiring vancomycin treatment. Because of the large doses of amines, he developed dry necrosis of both extremities. He also required transient pacing for extreme bradycardia. Hemorrhagic bronchial secretions caused several respiratory arrests. Finally, he developed a severe CMV colitis requiring sigmoidectomy and ganciclovir.

He stayed in ICU for 45 days and was released from hospital after 70 days. He received care for skin necrosis of the feet and he had a brief hospitalization to restore bowel continuity.

## 3. Discussion

Leptospirosis is usually encountered in developing regions, but is also reported in economically developed countries. It is certainly rare in Switzerland with fewer than 10 diagnosed cases per year. Up to 10% of leptospirosis infections induce multiple organ failure (MOF)—including kidney, liver, respiratory and circulatory failure, acute pancreatitis and/or rhabdomyolysis [1].

**Figure 1:**
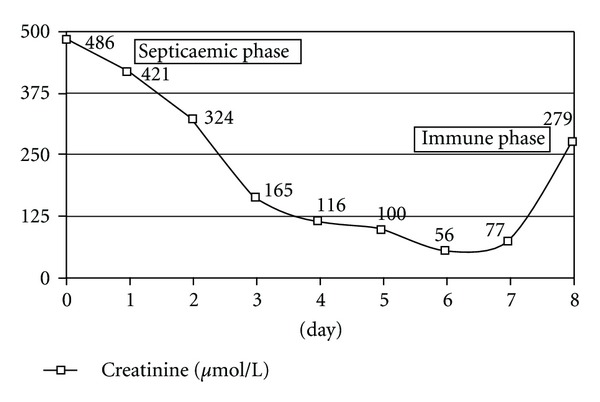
Evolution of creatinine mimicking the two phases of Weil's disease.

Leptospirosis is described as a biphasic illness characterized by two phases, an acute septicaemic phase followed by an immune phase (Figure 1). Most of exposed individuals develop mild symptomatic infection while a few progress to develop severe disease forms such as Weil's disease (the triad of jaundice, acute renal failure, and bleeding). The major burden of leptospirosis is due to Weil's disease with mortality higher than 50%. 

Leptospirosis causes a unique nonoliguric and hypokalemic form of acute kidney injury (AKI), hallmark features of impaired proximal sodium reabsorption. However, patients can develop oliguric AKI and even acute tubular necrosis associated with continued sodium and volume loss. The RIFLE criteria used to classify AKI in ICU and hospitalized patients have also been validated in AKI related to leptospirosis, with an incremental risk of mortality and dialysis requirement [4].

Clinical course and pathology of leptospirosis suggest an underlying immunopathogenic process and Jarisch-Herxheimer reaction is not an infrequent complication. This reaction occurs when large quantities of toxins are released into the body as bacterial death during antibiotic treatment. Typically release of endotoxins associated with the death of these bacteria occurs faster than the removal of the toxins by the body. Thus, Weil's disease is a classic model of sepsis. Hence, there is a pathophysiological reason for therapeutic plasma exchange in order to extract the excess of bacterial products and inflammatory mediators [1, 5]. Using the same rationale, some have reported success dialysis with albumin-molecular adsorbent recirculating system (MARS), in case of acute liver failure due to leptospirosis [6].

On the other hand, some studies have shown improved survival with high-volume hemofiltration in refractory septic shock [7, 8]. Therefore, in our unit, between 2007 and 2010, we do offer this treatment to all patients with refractory septic shock defined as a need for norepinephrine at a dose exceeding 0.2 *μ*g/kg/min. This is based on the recommendations of ADQI (acute dialysis quality initiative) group which emphasize that HVHF could be used by clinicians in catecholamine-resistant septic shock [9].

In the present case, we postulate that the immune phase resulted in multiple organ failure (MOF), despite early optimal medical management. Therefore, we decided to perform plasma exchanges followed by high-volume hemofiltration. After the implementation of these treatments, clinical and biochemical parameters significantly improved, suggesting that this therapeutical option was decisive for the patient survival and the complete recovery of renal and hepatic functions. However, the immunosuppression induced by plasma exchange may have facilitated another severe infectious complication, namely, CMV colitis requiring sigmoidectomy.

In conclusion, leptospirosis has complex clinical features including, in rare cases, severe renal and liver failure. The hypothesis of an immunological storm in this patient with severe refractory shock, prompted us to associate plasma exchange and high-volume hemofiltration successfully. Although we cannot determine in this case whether both treatments were mandatory lifesaving procedures, this therapeutic approach should be discussed in patients with leptospirosis, multiple-organ failure, and intractable septic shock.

## Figures and Tables

**Table 1:** Laboratory parameters at admission and during evolution of Weil's disease.

Parameter	At admission (day 0)	At Ward (day 5)	At ICU (day 8)	At Ward (day 45)	At end of stay (day 70)
Haemoglobin (g/L)	115	105	66	92	93
Thrombocytes (G/L)	22	69	301	177	172
TP (%)	>100		28	>100	
C-reactive protein (mg/L)	209	64	63	79	10
Potassium (mmol/L)	2.7	3.8	4.1	3.4	3.8
Bicarbonate (mmol/L)		26.2	8.1	23.8	
Urea (mmol/L)		13.6	27.7	15	5.9
*Creatinine (*μ*mol/L)*	*486*	*100*	*279*	*139*	*109*
CK (IU/L)	1730	66	353		
Troponin I (*μ*g/L)	0.118		0.17		
ASAT (IU/L)	185	113	660	37	41
ALAT (IU/L)	108	88	303	29	39
Bilirubin (*μ*mol/L)	285	522	726	25	18

## References

[1] Yang CW, Wu MS, Pan MJ (2001). Leptospirosis renal disease. *Nephrology Dialysis Transplantation*.

[2] Abdulkader RC, Silva MV (2008). The kidney in leptospirosis. *Pediatric Nephrology*.

[3] Andrade L, Cleto S, Seguro AC (2007). Door-to-dialysis time and daily hemodialysis in patients with leptospirosis: impact on mortality. *Clinical Journal of the American Society of Nephrology*.

[4] Basu G, Chrispal A, Boorugu H (2011). Acute kidney injury in tropical acute febrile illness in a tertiary care centre—RIFLE criteria validation. *Nephrology Dialysis Transplantation*.

[5] Tse KC, Yip PS, Hui KM (2002). Potential benefit of plasma exchange in treatment of severe icteric leptospirosis complicated by acute renal failure. *Clinical and Diagnostic Laboratory Immunology*.

[6] Covic A, Maftei ID, Gusbeth-Tatomir P (2007). Acute liver failure due to leptospirosis successfully treated with MARS (molecular adsorbent recirculating system) dialysis. *International Urology and Nephrology*.

[7] van Straaten HMO, Bosman RJ, van der Spoel JI, Zandstra DF (1999). Outcome of critically ill patients treated with intermittent high-volume haemofiltration: a prospective cohort analysis. *Intensive Care Medicine*.

[8] Honore PM, Jamez J, Wauthier M (2000). Prospective evaluation of short-term, high-volume isovolemic hemofiltration on the hemodynamic course and outcome in patients with intractable circulatory failure resulting from septic shock. *Critical Care Medicine*.

[9] Bellomo R, Honore PM, Matson J, Ronco C, Winchester J (2005). Extracorporeal blood treatment (EBT) methods in SIRS/Sepsis. *International Journal of Artificial Organs*.

